# Effects of a late supper on digestion and the absorption of dietary carbohydrates in the following morning

**DOI:** 10.1186/1880-6805-32-9

**Published:** 2013-05-25

**Authors:** Yukie Tsuchida, Sawa Hata, Yoshiaki Sone

**Affiliations:** 1 Early Childhood Education Department, Tokiwakai College, 4-6-7 Hirano-minami, Hirano-ku, Osaka 547-0031, Japan; 2 Graduate School of Human Life Science, Osaka City University, 3-3-138Sugimoto, Sumiyoshi-ku, Osaka 558-8585, Japan

**Keywords:** Suppertime, Carbohydrate absorption, Postprandial serum glucose profile, Breath hydrogen test

## Abstract

**Background:**

Our previous experiment showed that the light intensity exposed on the subjects during evening time had no effect in the following morning on the efficiency of the digestion and absorption of dietary carbohydrates ingested at a usual suppertime. People who keep late hours usually have a late suppertime; thus, we examined the effects of a late suppertime on gastrointestinal activity in the following morning in comparison to that of a usual suppertime.

**Methods:**

Twelve female university students volunteered as paid participants. The breath hydrogen test was carried out to estimate the amount of unabsorbed dietary carbohydrates and the percentage of the total amount of dietary carbohydrates in the breakfast that were unabsorbed, as well as to estimate oro-cecal transit time. The respiratory quotient was also measured to find the ratio of carbohydrates/lipid metabolism in the post-breakfast state. Subjects’ peripheral blood glucose concentration was measured by a blood glucose meter. The subjects participated under two different experimental conditions: with a usual suppertime (having supper at 18:00) and a late suppertime (having supper at 23:00).

**Results:**

The efficiency of the digestion and absorption of dietary carbohydrates in the breakfast under late suppertime conditions was higher than that under usual suppertime conditions. Usual or late suppertime had no effect on the ratio of carbohydrates to lipids oxidized after the subjects had breakfast. There were significant differences in the blood glucose level between the two conditions at 30, 60, 120, 150, and 180 minutes after having breakfast, whereas the mean blood glucose level under late suppertime conditions was significantly higher than under usual suppertime conditions.

**Conclusions:**

Having a late supper showed a worse effect on postprandial serum glucose profiles the following morning. This study confirmed that keeping our usual meal timing is important for our health.

## Background

Recently, young Japanese peoples’ lifestyle of keeping late hours means their suppertime is now occurring later in the evening [1,2]. This lifestyle of keeping late hours (a nocturnal lifestyle) is a worldwide trend and may be due to the recent rapid development of an artificial light environment at night. This nocturnal lifestyle is challenging modern human beings, especially those living in metropolitan areas, to adapt to a living environment that is artificially lit, which is unlike previous human experience. This new artificial light condition that recent human beings are confronted with may cause several modern diseases, and epidemiological surveys showed the nocturnal lifestyle is one of the causes of lifestyle-related diseases, especially of obesity [3]. Concerning the relationship between a nocturnal lifestyle and obesity, Nakamura *et al*. studied the effect of a late suppertime on energy metabolism during the hours of sleep and reported that postprandial energy consumption after having a late supper was lower than that after taking supper at the usual time [4]. In addition, Sekino *et al*. and Romon *et al*. studied the influences of eating rhythm on diet-induced thermogenesis in young women and reported that diet-induced thermogenesis for the night pattern meals was lower than that for the morning pattern meals [5,6]. Concerning to the effect of late meal on the obesity, Sato *et al*. reported that a single loading of late evening meal enhances average blood glucose over 24 hours, but does not reduce 24-hour energy expenditure [7]. These reports suggested that unusual eating rhythms, such as having supper late, have some effects on human energy metabolism, probably including the gastrointestinal activities.

During the course of our investigations on the effect of an artificially bright evening environment on human digestive physiology, we found that staying under a dim light from 17:00 to 02:00 after taking supper at 16:30 has a better effect on the digestion and absorption of dietary carbohydrates in the supper meal than staying under a bright light for the same period [8]. On the other hand exposure of the subjects to bright or dim light after they had supper at 17:30 in the evening (light conditions were controlled from 15:00 to 24:00) showed no different effect on the efficiency of digestion and absorption of dietary carbohydrates in the following morning meal [9]. This result indicated that the enhancement of sympathetic nervous tone induced by bright light exposure [10,11], which was supposed to be a major regulatory factor for gastrointestinal activity, ceased the following morning when participants were able to get sleep during the night. This result raises the question ‘does a late suppertime accompanied by a nocturnal lifestyle influence gastrointestinal activity including the efficiency of digestion and absorption of dietary carbohydrates in the following morning meal?’

A Survey on Time Use and Leisure Activities in 2006 [12] reported that about 60 percent of Japanese people started having supper before and around 19:00, while about 20 percent of people started having supper after 20:00 on weekdays. In relation to the younger generation’s lifestyle of having supper late in the evening [1,2], we found that overweight women who want to lose weight had a tendency to change their dietary habits. In the trial to make elderly women’s dietary life better, a dieting class had an effect on the participants, and their suppertimes were moved to earlier times than before the women participated in the class [13]. This implies that overweight people consider that keeping proper dietary habits, including a proper food intake rhythm, is important for keeping their proper weight and health. At present, however, we have no idea whether a late supper has a positive or negative effect on gastrointestinal activity in our everyday life. This means that we had no evidence to encourage overweight people to stay motivated enough to keep a proper food intake rhythm. These facts prompted us to examine the effect of a late supper on gastrointestinal activity the following morning by comparison of the efficiency of the digestion and absorption of dietary carbohydrates in the following breakfast under two experimental conditions; a usual suppertime (having supper at 18:00) and a late suppertime (having supper at 23:00).

## Methods

### Subjects

Twelve female university students volunteered as paid participants. Their characteristics are summarized in Table 1. All participants were non-smokers and they were required to report any current antibiotic therapy [14]. All subjects underwent the examination in the follicular phase of their menstrual cycles, because the menstrual cycle could affect their gastrointestinal activity [15]. We explained the purpose of the study and the procedure before their consent to participate in this study was given in writing. This was done according to the protocol approved by the Research Ethics Committee of the Graduate School of Human Life Science, Osaka City University (approval No. 10–16). This experiment was carried out in February and March, 2011.

**Table 1:** Subjects’ characteristics

Age (years)	21.3 ± 1.6
Height (cm)	159.1 ± 5.6
Weight (kg)	51.7 ± 4.4
BMI (kg/m^2^)	20.5 ± 2.1

Values are Mean ± SD.

### Experimental protocol and measurements

The breath hydrogen test and unabsorbed dietary carbohydrates (UDC: grams as lacto sucrose equivalent), percentage of unabsorbed carbohydrates in the total carbohydrates in the breakfast (% UDC) and oro-cecal transit time (OCTT) were calculated according to our previous studies [16,17]. Subject’s end-alveolar breath samples were collected every 20 min, beginning at 07:50 h, into special airtight bags using an Alveolar Gas Collection System AGC-3000 (Breath Lab Co. Ltd., Nara, Japan) and hydrogen concentrations were measured by gas chromatography (Breath Gas Analyzer model BGA-1000; Breath Lab Co. Ltd., Nara, Japan).

**Figure 1:**
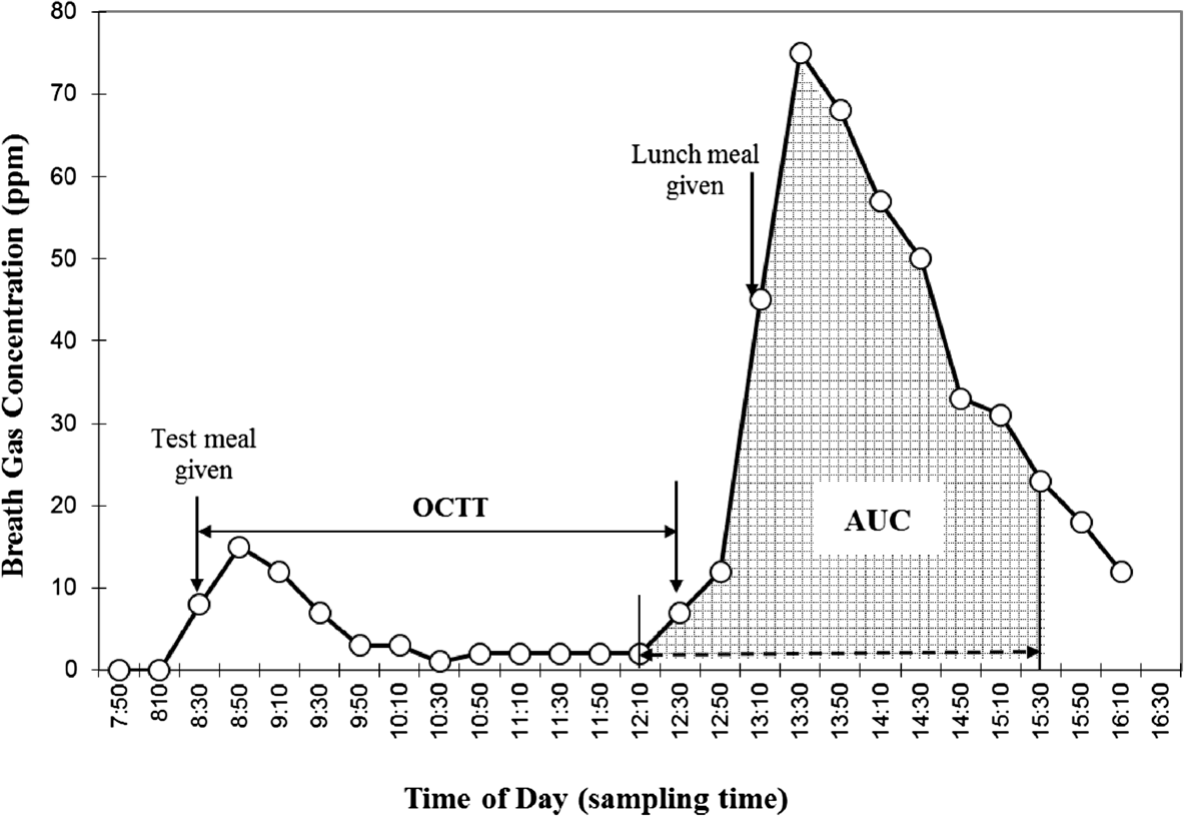
**A typical curve of hydrogen concentration versus breath sampling time of day following breakfast.** AUC, area under the curve; OCTT, oro-cecal transit time.

Figure 1 shows a typical curve of hydrogen concentration versus breath sampling time, where ‘hydrogen concentration’ (ppm) is the sum of the hydrogen and methane concentrations. Because it has been shown that there is a rough linear correlation between the excretion of hydrogen in the breath and the quantity of unabsorbed carbohydrate [18], the area under the curve when hydrogen concentration in the breath was plotted against time (abbreviated to AUC) was used to represent the amount of hydrogen resulting from fermentation of unabsorbed carbohydrate by the microflora of the large bowel. AUC was calculated according to the trapezoidal rule [19] and was expressed in the units of parts per million · hour (ppm · h). We defined AUC values corresponding to the breakfast meal or lacto sucrose solution as the area under the curve for 3 hrs and 20 min, commencing with the point 20 min before the rise in breath hydrogen level above the individual baseline value. This time of rise was defined as one of more than 5 ppm and one that was followed by at least two more rises [20]. This point was considered to be when the head of chyme entered the cecum. The length of time between 08:30 h and the time of rise (see above) was defined as the OCTT (expressed in minutes). The amount of unabsorbed dietary carbohydrate (UDC) from the breakfast meal was evaluated as the ‘lacto sucrose equivalent (g)’, calculated as follows:

$$6.2\times \left(\mathrm{AUC}\;\mathrm{for}\;\mathrm{the}\;\mathrm{breakfast}\;\mathrm{meal}/\mathrm{AUC}\;\mathrm{for}\;6.2g\;\mathrm{of}\;\mathrm{lactosucrose}\right)$$

The ratio of the UDC of the breakfast to its total carbohydrate content was calculated on the assumption that 4.9 g of dietary fiber in the breakfast are also fermented in the colon by its microflora [21]. Therefore, the percentage of the lack of intestinal digestion of the breakfast meal was calculated by the following equation:

$$\mathrm{UDC}\left(\mathrm{lacto}\;\mathrm{sucrose}\;\mathrm{equivalent},g\right)/68.4\times 100$$

The respiratory quotient (RQ) was measured by means of indirect calorimetry using the mixing chamber method with a Portable Gas Monitor AR-1 (type 4, Arco System, Chiba, Japan) connected to a facemask according to Morinaka *et al*. [22], and the RQ values during the period of stable breathing were used for further analysis.

Subjects’ peripheral blood glucose concentration was measured by a blood glucose meter with single-packed 3D Test Tips (TERUMO, Tokyo, Japan).

We estimated the whole gut transit time of breakfast according to subjects’ reports of when they observed black in their faces due to the squid ink in the breakfast spaghetti.

The test meal (the breakfast) was prepared from commercially available ready-to-eat minestrone and squid ink spaghetti (Table 2). The nutritional composition of all three meals provided on the day before the examination in this study is summarized in Table 3.

**Table 2:** Nutrient composition of the test meal for the examination (Days 2, 4)

Weight (g)	405
Energy (kcal)	406
Protein (g)	13.0
Fat (g)	5.8
Carbohydrate (g)	68.4
Dietary fiber (g)	4.9
Salt (g)	3.1

**Table 3:** Nutrient composition of the meal on the day before the examination (Days 1, 3)

	**Breakfast**	**Lunch**	**Supper**	**Total**
Weight (g)	530	682	455	1667
Energy (kcal)	570	670	690	1930
Protein (g)	18.1	29.7	23.2	71.0
Fat (g)	9.1	15.5	20.0	44.6
Carbohydrate (g)	85.0	99.2	101.2	285.4
Dietary fiber (g)	4.9	4.7	4.0	13.6
Salt (g)	2.6	3.3	2.3	8.2

**Figure 2:**
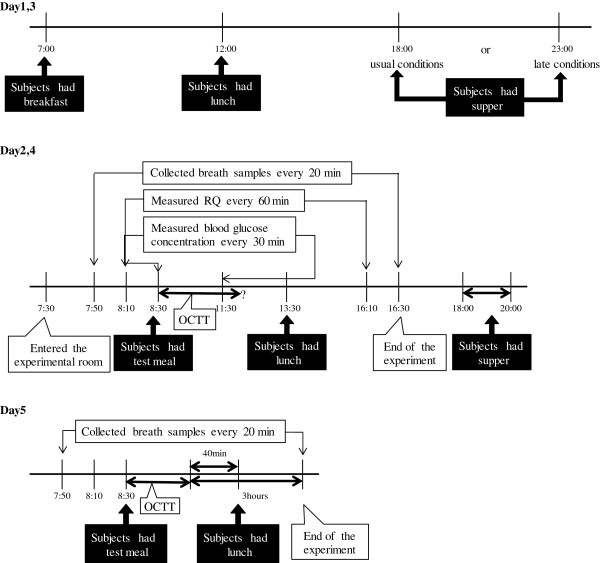
**Experimental protocol.** The assignment of the subjects to usual or late suppertime conditions was random for each subject. OCTT, oro-cecal transit time; RQ, respiratory quotient.

Figure 2 shows the experimental protocol of the examination. In this study, each subject had supper at 18:00 (usual suppertime conditions) or at 23:00 (late suppertime conditions) on the day before the examination. The examination included the breath hydrogen test, monitoring of RQ and peripheral blood glucose concentration and took place after breakfast. The assignment of the subjects to usual or late suppertime conditions was random for each subject. In order to control the subjects’ total energy intake and food items in the breakfast and lunch on the day before the examination, all subjects had the same kind of breakfast and lunch (Table 3) at 07:00 and 12:00, respectively, which was provided by the examiner two days before participation in the examination. Subjects were also required to retire by 24:00 on the nights prior to participation in the examination in order to get 6 or more hours of sleep. On the day of the examination, subjects entered the experimental room at 07:40 and their first end-alveolar breath sample was collected, the fasting RQ was measured for 15 min, and then the fasting blood glucose level was measured at 08:30. The subjects had breakfast around 08:30, and after that the breath hydrogen concentration, RQ value, and blood glucose levels were measured every 20 min, 60 min (until 16:30), and 30 min (until 11:30), respectively. Subjects had lunch around 13:30 after collection of an end-alveolar breath sample at 13:30. On day 5, the breath hydrogen test was carried out using an indigestible trisaccharide solution (200 ml of water containing 6.2 g of commercially available lacto sucrose, Ensuiko Co. Ltd., Shizuoka, Japan) as a meal in order to find the breath hydrogen production level.

### Data analysis

All data are shown as mean ± standard deviation (SD). Differences of mean OCTT, UDC, %UCD, and whole gut transit time for breakfast under the two suppertime conditions were assessed by Student’s paired *t* test. A comparison of the variation with time of RQ values and blood glucose levels after having breakfast under the two suppertime conditions was performed by the two factor (time and suppertime conditions) repeated measure analysis of variance (ANOVA). We used IBM SPSS ver.19.0 (SPSS Inc., Illinois) for the statistical analyses. A *P* value <0.05 was considered to be statistically significant.

## Results

Table 4 shows means and SD of OCTT (minutes), whole gut transit time (hours), UDC (gram, lacto sucrose equivalent) and the %UDC in the breakfast under the two suppertime conditions. The mean OCTT obtained under the late condition was significantly longer than that under the usual condition (*P* = 0.023), and UDC and %UDC under the usual condition were significantly higher than those under the late condition (*P* = 0.029, *P* = 0.016, respectively). These differences in UDC and %UDC indicated that the efficiency of digestion and absorption of the dietary carbohydrates in breakfast under the late suppertime condition was higher than that under the usual suppertime condition.

**Table 4:** Gastrointestinal activity values

	**Examination conditions**		
	**Usual conditions**	**Late conditions**	***P *value**^**a**^
OCTT (minutes)	278 ± 53^b^	315 ± 27^b^	0.023
UDC (g)	9.16 ± 7.78	5.48 ± 4.62	0.029
% UDC	12.6 ± 10.6	8.0 ± 6.8	0.016
Whole gut transit time (hours)	30.7 ± 16.4	34.4 ± 18.3	0.389

^a^Student’s paired *t* test.

^b^Values are Mean ± SD.

*OCTT* oro-cecal transit time, *UDC* unabsorbed dietary carbohydrates, *% UDC* percentage of unabsorbed dietary carbohydrates in the total carbohydrates in the breakfast.

**Figure 3:**
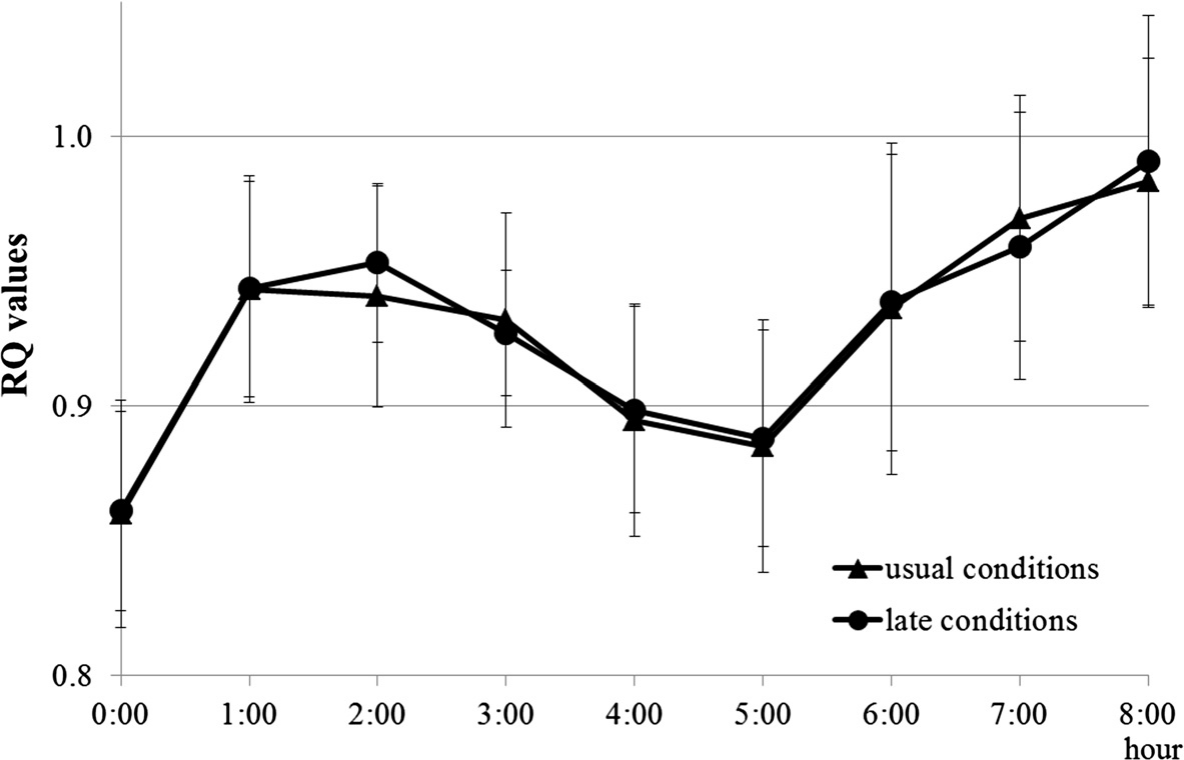
**Means of respiratory quotient (RQ) values (with SD bars) every hour before and after having breakfast.** SD, standard deviation.

Figure 3 shows the time course of the RQ values (mean with SD bars) after having breakfast (▲ - ▲, usual suppertime condition, ● - ●, late suppertime condition). Two factor repeated measures ANOVA showed that there was a significant main effect for time [*F*(8, 88) = 24.9, *P* = 0.000], while there was no significant difference between the two conditions [*F*(1, 12 ) = 0.024, *P* = 0.879], no significant interaction between the two factors (time, suppertime conditions) [*F*(8, 88) = 0.288, *P* = 0.968]. This result indicated that usual or late suppertime had no effect on the ratio of carbohydrates to lipids oxidized after having breakfast.

**Figure 4:**
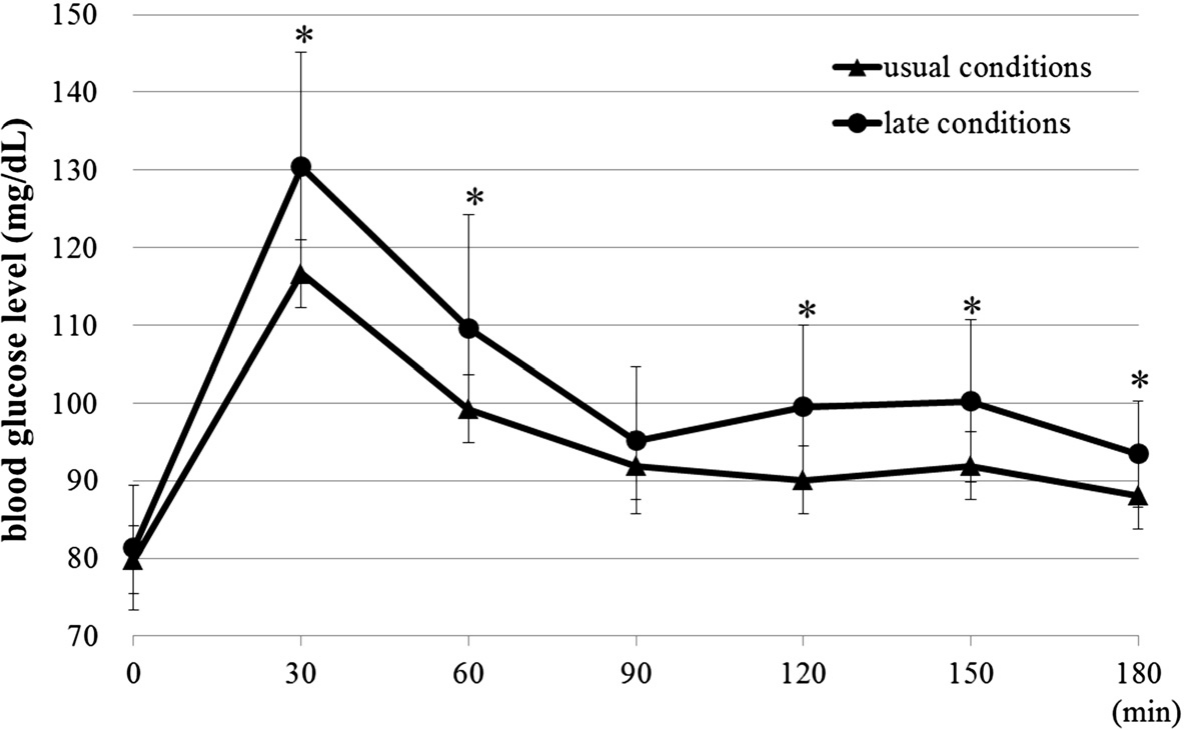
**Means of blood glucose level (with SD bars) every 30 minutes before and after having breakfast.** (*P <0.05 Student’s paired *t* test) SD, standard deviation.

Figure 4 shows the time course of the blood glucose level (mean with SD bars) after having breakfast (▲ - ▲, usual suppertime condition, ● - ●, late suppertime condition). Two factors repeated measures ANOVA showed that there was a significant main effect for time [*F*(6 , 66) = 48.781, *P* = 0.000], in addition, there was a significant difference between the two conditions [*F*(1 , 11) = 14.583, *P* = 0.003], but no significant interaction between the two factors (time, suppertime conditions) [*F*(6, 66 ) = 1.803, *P* = 0.112]. Student’s paired *t* test showed that there was a significant difference in the blood glucose level between the two conditions at 30 ,60, 120 , 150 and 180 min after having breakfast, where the mean blood glucose level under the late suppertime condition was significantly higher than that under the usual suppertime condition (*P* <0.05).

## Discussion

In this study, we aimed to find the effect of having supper late in the evening (at 23:00) on digestion and the absorption of dietary carbohydrates the following morning compared to that when having supper at a usual time (at 18:00). Concerning the effect of a light evening environment on human gastrointestinal activity, as mentioned in the introduction, Hirota *et al*. [8] reported that light intensity (dim or bright) in the evening (until 24:00) after taking supper (at 17:30) has no effect on gastrointestinal activity the following morning judging by the examination of the efficiency of digestion and the absorption of dietary carbohydrates in breakfast. In contrast to Hirota’s experiment, the present study showed that the oro-cecal transit time of chyme and the efficiency of carbohydrate absorption of breakfast the following morning were different between the two suppertimes the previous evening. Table 4 clearly shows that the efficiency of carbohydrate digestion and absorption obtained under the late suppertime condition was higher than that under the usual suppertime condition (the figures in the table show unabsorbed carbohydrates and their percentage in the whole carbohydrate content in the breakfast; therefore, the smaller the figures show the higher the efficiency of digestion and absorption of the dietary carbohydrates). This higher efficiency may be explained by the longer oro-cecal transit time of the chyme after a late supper intake than that after a usual supper intake. The longer oro-cecal transit time means that the chyme was exposed to digestion and the absorption tract for the longer period. Therefore, the efficiency of absorption of carbohydrates after having a late supper became better than that after having the meal at a usual suppertime [23]. This longer oro-cecal transit time and the higher efficiency of digestion and absorption of dietary carbohydrates resulted in higher blood glucose levels after having breakfast under the late suppertime condition than those under the usual suppertime condition. Figure 3 shows no significant difference in RQ values after having breakfast between the two suppertime conditions indicating no reduction in carbohydrate oxidation under the late suppertime condition. This ruled out the possibility that a reduced carbohydrate metabolic rate resulted in higher blood glucose levels after breakfast under the late suppertime condition. The phenomena observed in the present study (the slower motility of the gastrointestinal tract and the higher blood glucose levels in the postprandial state) were very similar to those observed in the experimental euglycemic hyperinsulinemia subjects reported by Eliasson *et al*. [24]. They observed and reported that experimental euglycemic hyperinsulinemia induced a significant delay of postprandial gastric emptying and resulting higher blood glucose levels after food ingestion. They also reported that the blood levels of the motility-stimulating hormone (motilin) were significantly low during the experimental hyperinsulinemia. In this connection, Kaneko *et al*. [25] reported that subjects who led a nocturnal lifestyle, where they skipped breakfast and got most of their energy from a late supper, for a week showed higher insulin levels in the evening and the following morning (18:00 to 6:00) than those who led a normal lifestyle. In addition Nakamura *et al*. [5] reported that they observed elevated energy expenditure, increased heart rate, and higher blood glucose levels during sleeping hours after a late supper, and they suggested that those resulted from higher insulin levels at night and during sleep. These reports described above may postulate the hypothesis that a relatively higher insulin level was induced by the late supper and this situation lasted until the following morning and resulted in hyperinsulinemia, which made the oro-cecal transit time of breakfast longer and blood glucose levels higher after breakfast than that under a usual suppertime condition. Of course we need further experiments to prove this hypothesis by measuring blood insulin and motilin levels the morning following a late supper.

In this experiment we observed significantly higher blood glucose levels for 3 hours after breakfast under the late suppertime condition compared to that under the usual suppertime condition (Figure 4). The higher blood glucose levels in the postprandial stage (post-meal hyperglycemia) have been pointed out as a risk factor for diabetes related diseases because many clinical trials have demonstrated no glycemic threshold for induction of either microvascular or macrovascular complications; the higher the glycated hemoglobin (HbA1c), the higher the risk for diabetes [26]. In addition Morgan *et al*. reported meal timing affected glucose tolerance and insulin secretion and recommended avoidance of large meals in the evening for improving postprandial glucose profiles [27]. These imply that habitual late suppers may induce chronic diabetes related diseases. Therefore, this study may help overweight people to consider keeping proper dietary habits including a proper food intake rhythm important for maintaining their proper weight and health.

## Conclusions

Having a late-supper showed the non-beneficial effect on postprandial glucose profiles the following morning. This study confirmed that maintaining regular (early) meal timing is important for our health.

## Abbreviations

OCTT: Oro-cecal transit time; RQ: Respiratory quotient; SD: Standard deviation; UDC: Unabsorbed dietary carbohydrates; %UDC: Percentage of unabsorbed dietary carbohydrates in the total carbohydrates in the breakfast.

## Competing interests

The authors have no conflict of interest to declare.

## Authors’ contributions

YS did the study design, was general coordinator. YT were designed the study, involved in data collection, data interpretation and result analysis and literature search. SH were involved in data collection, data interpretation. All authors read and approved the final manuscript.
